# SARS-CoV-2 in peritoneal swabs from asymptomatic patients undergoing emergency abdominal surgery

**DOI:** 10.1093/jscr/rjab116

**Published:** 2021-04-09

**Authors:** Jasim AlAradi, Rawan A Rahman AlHarmi, Mariam AlKooheji, Sayed Ali Almahari, Mohamed Abdulla Isa, Raed AlMarzooq

**Affiliations:** Department of Surgery, Salmaniya Medical Complex, Manama, Bahrain; Department of Surgery, Salmaniya Medical Complex, Manama, Bahrain; Department of Surgery, Salmaniya Medical Complex, Manama, Bahrain; Department of Pathology, Salmaniya Medical Complex, Manama, Bahrain; Department of Surgery, Salmaniya Medical Complex, Manama, Bahrain; Department of Surgery, Salmaniya Medical Complex, Manama, Bahrain

## Abstract

This is a case series of five patients with acute abdomen requiring surgery who tested positive for coronavirus disease 2019 (COVID-19) and were asymptomatic, with the purpose of detection of severe acute respiratory syndrome coronavirus 2 (SARS-CoV-2) in peritoneal fluid. Nasopharyngeal swab was done as a prerequisite for admission or prior to admission as part of random testing. Two methods of viral testing were employed: Xpert® Xpress SARS-CoV-2 (rapid test) and real-time reverse transcription polymerase chain reaction (RT-PCR). Either or both tests were done, with the former performed for patients requiring surgery immediately. Surgery was performed within 24–36 h from admission. Peritoneal fluid swabs were obtained for the detection of SARS-CoV-2 using RT-PCR test. Swabs were immediately placed in viral transfer media and delivered to the public health laboratory in an ice bag. SARS-CoV-2 was not detected in peritoneal swabs. Due to the limited number of patients, further studies are required; yet, protective measures should still be taken by surgeons when dealing with COVID-19 cases.

## INTRODUCTION

Coronavirus disease 2019 (COVID-19) is caused by the novel coronavirus named severe acute respiratory syndrome coronavirus 2 (SARS-CoV-2). Emerging late in December 2019, the disease presents most commonly with fever, cough and often severe respiratory syndrome [1]. The main route of transmission is through inhalation of infectious aerosol. It is unknown whether inhalation of surgical smoke generated during open and laparoscopic surgery causes transmission of the virus. In addition, healthcare workers exposed to surgical patients might be at increased risk due to the close and prolonged contact with the patient [2].

The virus has been detected in samples including nasopharyngeal swabs, sputum, alveolar lavage fluid, blood, stool and saliva, among others [1, 3]. We aim through this series to detect SARS-CoV-2 in peritoneal fluid from COVID-19-positive patients undergoing emergency abdominal surgery.

## CASE SERIES

This is a case series of five patients admitted to our facility under General Surgery with acute abdomen requiring an emergency surgery who tested positive for COVID-19 and were asymptomatic. Nasopharyngeal swab was done as per hospital protocol as a prerequisite for admission and surgery or done prior to admission as part of random testing of citizens and residents. Two methods of molecular viral testing are available: Xpert® Xpress SARS-CoV-2 (real-time, rapid test) and real-time reverse transcription polymerase chain reaction (RT-PCR) [4]. Cycle threshold (Ct) <40 is considered positive [5]. Targets are envelope (E) and nucleocapsid (N2) genes. Patients had either or both tests done. Rapid tests were usually performed for patients requiring surgery immediately. All patients were allocated to isolation wards. Patients underwent surgery within 24–36 h from admission in a negative pressure operating theater. Peritoneal fluid swabs were obtained for the detection of SARS-CoV-2, on which RT-PCR test was performed. Swabs were immediately placed in viral transfer media and delivered to the public health laboratory in an ice bag. Patient data was collected from the National Health Information System (I-SEHA). Ethical approval was attained.

Table 1 summarizes the data of the patients. They are numbered according to the chronological order of presentation. All patients presented to the emergency department with the chief complaint of abdominal pain, four of which had right lower quadrant pain suggestive of acute appendicitis, and one presented with right inguinal pain of sudden onset diagnosed with incarcerated hernia. The latter was diagnosed with COVID-19 through random testing of citizens and residents just prior to presenting to the emergency department. The rest were diagnosed through nasopharyngeal swab done as per hospital protocol as a prerequisite for admission and surgery. None of the patients complained of respiratory symptoms upon presentation. None had documented contact with positive patients either. Chest radiographs were done for most of the patients upon admission. Case 3, incarcerated right inguinal hernia, had an initial acceptable chest radiograph; however, he later developed pneumonia through his hospital course. Patients were admitted in COVID-19 wards. All patients were discharged home after appropriate treatment.

**Table 1:** Summary of cases

Case	Sex	Age (years)	Comorbidities	Admitting diagnosis	Imaging employed	Reason for swab	Nasopharyngeal swab (type of test)	Ct value	Surgery performed and timing	Operative findings	Peritoneal swab for culture and sensitivity	Peritoneal swab (RT-PCR)	Histopathology	COVID-19 complications
1	Male	34	None	Acute appendicitis	Ultrasound abdomen	Admission	Xpert® Xpress SARS-CoV-2 [Rapid]	Not specified	Open appendectomy on 2nd day of admission	Acutely inflamed appendix	Sterile	Negative	Acute suppurative appendicitis	None
2	Male	25	None	Acute appendicitis	Ultrasound abdomen	Admission	Xpert® Xpress SARS-CoV-2 [Rapid], RT-PCR	Ct 30	Open appendectomy on 1st day of admission	Acutely inflamed appendix	Sterile	Negative	Acute appendicitis	None
3	Male	55	Diabetes mellitus, hypertension	Incarcerated right inguinal hernia	Clinical diagnosis	Random testing of citizens and residents	RT-PCR	Ct 28.7	Exploration and repair of hernia on 1st day of admission	Indirect hernia with bowel content and fluid in sac	Not done	Negative	Hernial sac: unremarkable	Pneumonia (resolved)
4	Male	22	None	Acute appendicitis	Ultrasound abdomen	Admission	Xpert® Xpress SARS-CoV-2 [Rapid]	Ct-E 21.4 Ct-N2 23.1	Open appendectomy on 1st day of admission	Acutely inflamed appendix	Sterile	Negative	Acute suppurative appendicitis	None
5	Male	35	None	Acute appendicitis	CT abdomen	Admission	Xpert® Xpress SARS-CoV-2 [Rapid]	Ct-E 21.8 Ct-N2 24.4	Open appendectomy on 1st day of admission	Acutely inflamed appendix	Sterile	Negative	Acute suppurative appendicitis	None

## DISCUSSION

Several studies were published recently since the emergence of COVID-19, addressing the possibility of detection of its causative agent, SARS-CoV-2, in different body fluid samples, including peritoneal fluid, and its implications on healthcare workers, particularly surgeons.

Ngaserin *et al*. [6] report a case of acute appendicitis who underwent laparoscopic appendectomy, in whom SARS-CoV-2 was not detected in peritoneal fluid and washings. Similarly, Flemming *et al*. [7] report a case of a critically ill patient who required an emergency cholecystectomy, in whom ascitic fluid, bile, liver and gallbladder samples were collected and were all negative for SARS-CoV-2. In line with the previous studies, Vudayagiri and Gusz [8] report a case of acute appendicitis who underwent laparoscopic appendectomy, in whom SARS-CoV-2 was detected in the nasopharyngeal swab but not in the peritoneal fluid sample. Also, Seeliger *et al*. [9] report five positive cases requiring emergency abdominal surgeries and none of them had positive peritoneal swabs. These results are similar to our findings.

On the other hand, a number of authors reported detection of the virus in peritoneal samples. In a paper by Vischini *et al*. [10], peritoneal dialysate of a patient with fibrillary glomerulonephritis and end-stage kidney disease on peritoneal dialysis was positive for SARS-CoV-2. Additional supporting findings are reported by Rimini *et al*. [11] as they describe a case of incarcerated hernia who underwent exploratory laparotomy, in whom the virus was detected in the peritoneal swab. Likewise, Culver *et al*. [12] report a case of a patient with upper gastrointestinal bleeding and cirrhosis, in whom blood and ascitic fluid were positive for the virus. Moreover, Barberis *et al*. [13] report a case of lower gastrointestinal bleeding who required subtotal colectomy with terminal ileostomy who had a positive peritoneal swab. Lastly, Coccolini *et al*. [14] describe a case of mechanical small bowel obstruction due to volvulus with COVID-19 pneumonia who underwent exploratory laparotomy with adhesiolysis and SARS-CoV-2 was detected in peritoneal fluid, interestingly, at a higher concentration than in the respiratory tract. These results are important to consider when dealing with surgical patients as this might pose risk of contagion to the operating surgeon and other personnel.

## CONCLUSION

In our series of cases and in line with other studies, SARS-CoV-2 was not detected in swabs obtained from peritoneal fluid in patients undergoing emergency abdominal surgery with positive nasopharyngeal swabs. Due to the limited number of patients, further studies are needed to draw better conclusions. Yet, protective measures should still be taken by operating surgeons when dealing with COVID-19-positive cases.

## SOURCE(S) OF SUPPORT

None declared.

## PRESENTATION AT A MEETING

This paper was not presented before in any meeting.
